# Leadless pacemaker implantation after transcatheter lead extraction in complex anatomy patient

**DOI:** 10.1002/ccr3.1532

**Published:** 2018-04-19

**Authors:** Valentina De Regibus, Antonino Pardeo, Paolo Artale, Andrea Petretta, Pasquale Filannino, Saverio Iacopino

**Affiliations:** ^1^ GVM Care&Research Maria Cecilia Hospital Cotignola Italy; ^2^ GVM Care&Research Città di Lecce Hospital Lecce Italy; ^3^ GVM Care&Research Anthea Hospital Bari Italy

**Keywords:** Dextrocardia, leadless pacemaker, situs viscerum inversus, transvenous lead extraction

## Abstract

After transvenous lead extraction, leadless pacemaker might be a valid alternative to the traditional two‐step strategy including an active fixation leads temporary PM and subsequent contralateral permanent implantation in patients who are pacemaker‐dependent. Moreover, leadless PM might be of great importance in patients presenting with congenital vascular or cardiac abnormality.

## Introduction

Recently, major advancements have been achieved in the leadless pacemaker technology development, leading to a device with similar efficacy and safety but lower infective risk than traditional one. We describe the case of a patient presenting dextrocardia and endocarditis successfully implanted with a leadless pacemaker after transvenous lead extraction.

Permanent cardiac pacing delivered by conventional pacemaker (PM) is the cornerstone in the treatment of bradycardia 1. Despite the reduction in complications due to technological developments, serious adverse events can still be encountered 2. In recent years, major advancements have been achieved in the leadless PM technology development, leading to a device that has shown efficacy and safety compared with the traditional PM 3. Nevertheless, the experience with such technology is limited.

We describe the successful but complex case of a patient presenting with dextrocardia implanted with a leadless pacemaker Medtronic Micra transcatheter pacemaker system (Micra).

## Case Report

**Figure 1:**
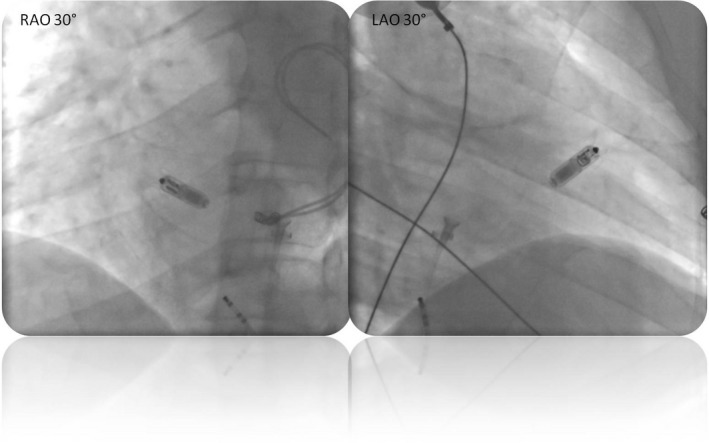
Right anterior oblique (RAO) and left anterior oblique (LAO) projections of the final position of Micra system in the heart.

A 36‐year‐old man presented with situs viscerum inversus and dextrocardia underwent permanent VVI pacemaker implantation in 1998 because of complete atrioventricular (AV) block. In 2006, the battery was replaced electively, while in 2007, lead extraction due to failure in capturing the right ventricle (RV) was performed and a dual chamber pacemaker was implanted and connected to an epicardial RV lead and to a right atrial lead intravenously implanted via left subclavian vein. The follow‐up was complicated by three surgical pocket revisions due to pocket decubitus. In May 2016, he experienced endocarditis that required transvenous lead extraction (TLE) of the atrial lead and extraction of the can, while the epicardial lead was abandoned in place. As the patient was PM‐dependent, a temporary PM via left femoral vein was placed before the extraction and left in place until a Micra was successfully implanted via the right femoral vein the same day. The electrical measurements were tested 10 times in different positions in order to get the best one. After the first seven attempts, despite a low bolus of 2500 IU of heparin delivered intravenously following placement of the introducer and the continuous cleaning with heparinized saline drip 4, the Micra delivering system was almost completely obstructed by clots; hence, we had to replace it with a new system. Finally, the Micra was successfully positioned in the anteroseptal region (Fig. 1). The final pacing threshold was 0.38 V/0.24 msec and sensing was 12.4 mV. The total procedure duration was 119 min, while the total fluoroscopic duration was 35 min. On a 12‐month follow‐up, the electrical measurements were stable and the Micra position was confirmed by chest X‐ray. No adverse events were reported.

## Discussion

We described a case report in which the use of Micra was a suitable option because of the device‐related endocarditis and the anatomical issue. According to TLE consensus 5, after the device removal, it is important to reassert the patient's indication for pacing and defibrillation. In case of persisting indication, a new contralateral implantation has to be planned after antibiotic therapy. In patients who are pacemaker‐dependent, an epicardial approach might be considered, especially in vascular or cardiac anatomical variants. Micra is a miniaturized single‐chamber pacemaker with electrodes being directly placed on the pacemaker capsule. This eliminates the need for a device pocket and insertion of a pacing lead, thereby eliminating an important source of complications associated with traditional pacing systems while providing similar benefits 6. For those reasons, Micra can be an alternative reimplantation strategy after endocarditis, giving the possibility to implant it immediately after the TLE in case of need. Krypta et al. 7 recently reported successful Micra implantation in pacemaker‐dependent patients who underwent TLE because of severe device infection.

Moreover, Micra might be of great importance in patients presenting with congenital vascular or cardiac abnormality. It has already been proven useful in one patient presenting with persistent superior vena cava and device‐related infection 8. Here, we described Micra implantation in a patient presenting with congenital heart abnormality resulting in dextrocardia, complete AV block requiring PM complicated by device‐related infection. In this rare and complex situation, Micra might be an important tool to avoid further more invasive procedures, as the epicardial approach. In particular, in the case that we presented, the patient already had an epicardial lead implantation. In addition, as the patient was pacemaker‐dependent, the need for an immediate pacing solution has been satisfied with Micra, instead of the traditional two‐step strategy including an active fixation leads temporary PM and subsequent contralateral permanent implantation 5. Micra allowed to perform a potentially easier procedure than contralateral implantation and potentially reducing the infective risk. In fact, although implanted without waiting for adequate antibiotic therapy period, Micra is similar in size to the lead's fragments that are occasionally left in place in RV after lead extraction and that seem to be associated with low rate of infection's recurrence 9.

## Conclusions

Micra seems to be a valuable alternative in patients with recurrent device‐related infections, especially in those with rare anatomies or previous multiple implantations.

## Conflict of Interest

None declared.

## Authorship

VDR: collected information on the patient, drafted the manuscript, and approved the final version of manuscript. AP: helped in drafting the manuscript and revised the contents of the discussion of the manuscript. PA: helped in drafting the manuscript and revised the contents of the discussion of the manuscript. AP: helped in drafting the manuscript and revised the contents of the discussion of the manuscript. PF: performed PM implantation, helped in drafting the manuscript, and revised the contents of the discussion of the manuscript. SI: performed PM implantation, helped in drafting the manuscript, and revised the contents of the discussion of the manuscript.
